# A Hypertrophic Stimulus Drives Age‐Dependent Transcript Isoform Switching With and Without Differential Gene Expression in Skeletal Muscle

**DOI:** 10.1111/acel.70761

**Published:** 2026-10-14

**Authors:** Pieter J. Koopmans, Abbey L. Stokes, Christopher E. Nelson, Yuan Wen, Ahmed Ismaeel, Kevin A. Murach

**Affiliations:** ^1^ Cell and Molecular Biology Graduate Program University of Arkansas Fayetteville Arkansas USA; ^2^ Molecular Muscle Mass Regulation (M3R) Laboratory, Department of Health, Human Performance, & Recreation University of Arkansas Fayetteville Arkansas USA; ^3^ Department of Biomedical Engineering University of Arkansas Fayetteville Arkansas USA; ^4^ Department of Physiology, College of Medicine University of Kentucky Lexington Kentucky USA; ^5^ Department of Internal Medicine, College of Medicine University of Kentucky Lexington Kentucky USA; ^6^ Department of Anatomy, Physiology, and Pharmacology, College of Veterinary Medicine Auburn University Auburn Alabama USA; ^7^ NextGen Precision Health Initiative University of Missouri Columbia Missouri USA; ^8^ Department of Nutrition and Exercise Physiology University of Missouri Columbia Missouri USA; ^9^ Department of Medical Pharmacology and Physiology University of Missouri Columbia Missouri USA

## Abstract

Aged skeletal muscle has a reduced ability to adapt to hypertrophic stimuli concomitant with dysregulation across several molecular layers. Whether altered RNA splicing associates with impaired adaptive potential is not well studied. We hypothesized that aging disrupts the splicing response to involuntary muscle mechanical loading. Using deep short‐read Illumina RNA‐sequencing (SRS, 60 million read pairs) and third generation Oxford Nanopore long‐read RNA‐sequencing (LRS) combined with a murine model of acute muscle mechanical overload (MOV), we illustrate how aged muscle has a reduced or possibly delayed potential for isoform switching in response to stress. SRS identified hundreds of genes with isoform switching events in adult MOV relative to sham but only 37 in aged MOV. LRS identified 19 genes (including *Cacnb1, Naca, Psap*) with true isoform switching in adult MOV and none in aged MOV. A subset of genes showed significant isoform switching without corresponding changes in overall gene expression, suggesting post‐transcriptional regulation in response to MOV. These data highlight the importance of analyzing the transcriptome at the isoform level to uncover transcript diversity and “unseen genes” that may have unique functions and mechanisms influencing hypertrophic adaptations.

## Graphical abstract

A combined analysis of short‐read and long‐read RNA‐seq demonstrates reduced isoform switching after an acute hypertrophic stimulus in aged muscle and identifies age‐dependent isoforms that may not be detected with total gene‐level analyses.

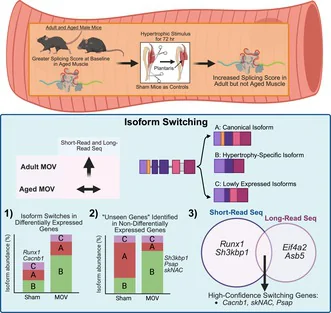

Aging as well as mechanical loading of skeletal muscle induce significant alterations to the transcriptome (Koopmans et al. 2026; Graber et al. 2023; Murach et al. 2022). A proposed hallmark of aging is dysregulation of alternative splicing (AS), a common post‐transcriptional mechanism that generates multiple protein variants from a single gene (Tumasian et al. 2021; Deschênes and Chabot 2017; García‐Ruiz et al. 2025). Alterations to AS during aging may in part explain the age‐related decline in muscle mass and adaptability (Tumasian et al. 2021). Using our recently published bulk RNA‐seq data, we show a number of genes in the KEGG spliceosome pathway are upregulated to a greater degree or exclusively in adult but not aged muscle during mechanical overload (MOV, Figure 1A). The overall “splicing score” was also higher during adult MOV but not aged (Figure 1B), with each module within the splicing pathway responding to a lesser degree in aged MOV compared to adult (Figure 1B'). Worth noting, the splicing score of aged sham muscle was greater than adult sham, which is consistent with prior work showing that alternative splicing is dysregulated with aging (Donega et al. 2026; Ferrucci et al. 2022; Ubaida‐Mohien et al. 2019). These observations motivated us to perform splicing analyses. We paired high‐depth short read Illumina RNA‐seq and Oxford Nanopore long‐read RNA‐seq obtained from the plantaris muscles of adult (6–8 month) and aged (24‐month) mice. We focused on differential isoform usage and differential isoform expression analyses after 72 h of MOV, or the “acute” phase of a hypertrophic stimulus prior to differential age‐dependent responses in growth (Dungan et al. 2022).

**FIGURE 1:**
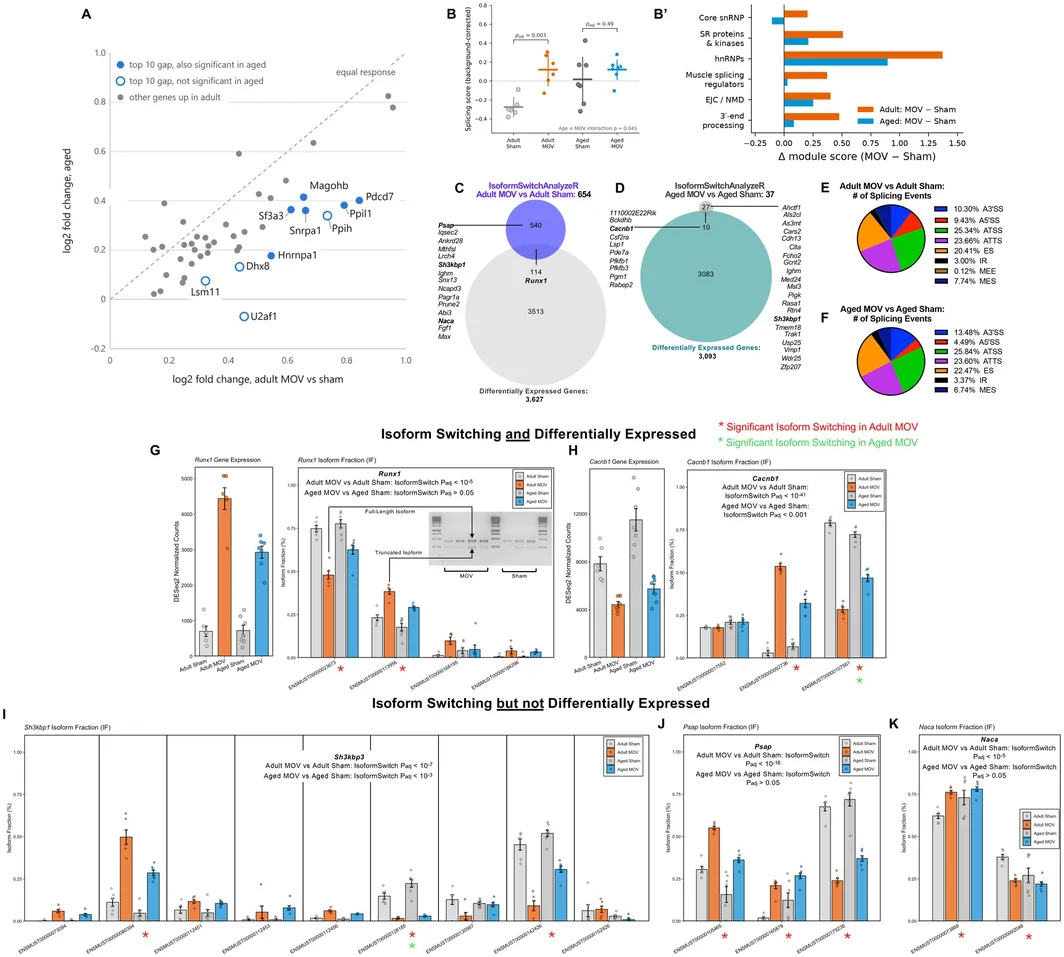
Analysis of Isoform Switching Events in Short‐Read RNA‐Seq. (A) Fold‐changes of genes in the KEGG spliceosome pathway altered during MOV with SRS, (B) Overall splicing score of each experimental condition, (B') Splicing score of each module in the spliceosome pathway. (C) Venn diagram comparing number of isoform switching genes with adult MOV vs. differentially expressed genes (DEG, Koopmans et al. 2026; *Advanced Science*). (D) Venn diagram comparing number of isoform switching genes with aged MOV versus DEG. Genes discussed in main text are in bold. (E and F) Pie chart showing number and proportion of isoform switching events with adult and aged MOV, respectively. (G) Example of a gene (*Runx1*) significantly upregulated in MOV driven by a significant abundance increase of multiple isoforms. (H) Example of gene (*Cacnb1*) significantly downregulated in MOV driven by a significant abundance decrease of one isoform. (I–K) Examples of genes with significant isoform switching events, despite showing no significant changes in total gene expression: *Sh3kbp1* (panel I), *Psap* (Panel J), *Naca* (Panel K). Alternative 3′ splice site (A3), alternative 5′ splice site (A5), alternative transcription start site (ATSS), alternative transcription termination site (ATTS), exon skipping (ES), intron retention (IR), mutually exclusive exons (MEE), and multiple exon‐skipping (MES). Error bars are mean ± SEM. SRS, *n* = 6–7 mice per condition. Differentially expressed gene defined as *p*‐adj < 0.05 and |Log_2_FC| ≥ 1.

## 1 Short‐Read RNA‐Sequencing Analysis Shows Mechanical Overload‐Induced Isoform Switching is Reduced with Aging

Short‐read (150 bp, 60 million read pairs per sample, *n* = 6–7 per group) Illumina RNA‐sequencing (SRS) was performed on RNA isolated from 72 h MOV or sham control plantaris muscles, as previously described (Koopmans et al. 2026; Murach et al. 2022). Salmon quantified transcript abundance matrices were imported into RStudio, and IsoformSwitchAnalyzeR workflow was used to identify genes with isoform‐switching events with an absolute difference in isoform fraction > 10% and *p*‐adj < 0.05 (Vitting‐Seerup and Sandelin 2019). With aging alone (aged sham vs. adult sham), 75 genes showed significant isoform switching (Figure S1A). In adult MOV, we detected 654 genes with significant isoform switching (Figure 1C) relative to adult sham, but only 37 in aged MOV relative to aged sham (Figure 1D). Of these isoform switching genes with adult MOV, 114 were identified as differentially expressed genes (DEGs, all isoforms together) in previously published RNA‐seq of these same samples (Koopmans et al. 2026). For aged MOV, 10 isoform switching genes also were DEGs. The most common mechanism leading to isoform switching with MOV in both ages was alternative transcription start site (ATSS, ~25% of events), alternative transcriptional termination site (ATTS, ~23% of events), and exon skipping (ES, 20%–22% of events, Figure 1E,F).

## 2 Isoform Switching in Genes with Differential Expression

In some cases, gene‐level differential expression results from coordinated changes of multiple transcript isoforms of the same gene (DEG defined as *p*‐adj < 0.05 and |Log_2_FC| ≥ 1). For example, Runt‐related transcription factor 1 (*Runx1*), which can regulate ribosome biogenesis, prevent myofibrillar disorganization, and attenuate atrophy during muscle denervation (Cai et al. 2015; Wang et al. 2005; Bao et al. 2018), is driven by upregulation of two isoforms in both ages during MOV; yet, only adult MOV showed significant isoform switching (*p*‐adj = 1.1 × 10^−6^, Figure 1G). There was a slight shift in isoform fraction (IF) from the longer canonical isoform (ENSMUST00000023673, 75%–46% IF) to a shorter non‐canonical isoform (ENSMUST00000113956, 23%–37% IF) lacking exons 1, 2, and 6. Exon 6‐null isoforms have enhanced protein stability and synergize with the exon 6‐positive isoform to enhance promoter transactivation (Komeno et al. 2014). The truncated isoform potentially has a unique function during muscle hypertrophy. As an example, to validate the presence of these isoforms, we performed PCR with primers specific for each isoform followed by gel electrophoresis. This analysis confirmed that both isoforms were present, and the short isoform was qualitatively more abundant during MOV (Figure 1G). Another example of a DEG with isoform switching was Calcium voltage‐gated channel auxiliary subunit beta 1 (*Cacnb1* or CaVβ1). *Cacnb1* was downregulated at the total gene level (Figure 1H) but featured among the most substantial isoform switching events in both ages (*p*‐adj < 0.001). In sham conditions, ENSMUST00000107561 was 72%–78% of IF but was substituted by greater abundance of ENSMUST00000092736 during MOV.

## 3 Significant Isoform Switching in Genes Without Differential Expression

In other cases with aging and MOV, there was isoform switching without gene‐level differential expression (defined as *p*‐adj < 0.05 and |Log_2_FC| > 1), highlighting the need to evaluate genes at the transcript usage level. With aging compared to adult (sham comparisons), *Tsc1, Champ1, Map4k5, and Hk2* showed isoform switching without differential gene expression (Figure S1B–E). TSC1 is a well‐characterized inhibitor of the mTORC1 signaling pathway, and Hexokinase 2 (HK2) is a critical enzyme in glucose metabolism. The functions of these non‐canonical isoforms in skeletal muscle are unknown (Wan et al. 2006; Roberts and Miyamoto 2015). During MOV, SH3‐domain Kinase Binding Protein 1 (*Sh3kbp1*), an endoplasmic reticulum scaffolding protein, was not altered at the total gene level with adult MOV, but showed a ~36%–38% isoform switch (*p*‐adj = 1.3 × 10^−8^) from ENSMUST00000142426 (non‐protein coding) to ENSMUST00000080394 (SH3‐domain containing). Most other transcripts were unchanged (Figure 1I). This shift in isoform fraction is qualitatively less pronounced in aged versus adult muscle during MOV (only 19%–24% isoform switch). SH3KBP1 has multifaceted roles in skeletal muscle; it appears critical for myonuclear organization during myofiber formation *in vitro* and is necessary for myonuclear movement in mature myotubes (Guiraud et al. 2025). Further, it regulates aspects of T‐tubule formation, excitation‐contraction coupling, and autophagy (Guiraud et al. 2025). Likewise, Prosaposin (*Psap*, Figure 1J) and Nascent Polypeptide‐Associated Protein Complex Subunit Alpha (*Naca*, Figure 1K) were not DEGs (Koopmans et al. 2026), but showed significant isoform switching in adult but not aged MOV mice (*Psap:* *p*‐adj< 1.96 × 10^−19^; *Naca*: *p*‐adj  < 7.1 × 10^−6^). The isoform switch in *Naca* involves the inclusion of a large exon, producing the skeletal muscle‐specific transcription factor variant (skNAC). skNAC functions as a ribosomal chaperone protein by coordinating assembly and transport of newly synthesized proteins to specific intracellular compartments. skNAC is also among the most highly phosphorylated proteins during acute aerobic and resistance exercise (Yotov and St‐Arnaud 1996; Berger et al. 2012; Pataky et al. 2025). It is conceivable that the inability to switch to this variant may contribute to blunted muscle adaptability in aged muscle given its phosphorylation appears important for exercise adaptation (Pataky et al. 2025). These examples demonstrate cases of transcript isoform switching without differential expression that would not have otherwise been detected by gene level analyses alone (Oakes and Pachter 2026).

## 4 Oxford Nanopore Long‐Read Sequencing Confirms Attenuated Isoform Switching with Aging

SRS sometimes misses low frequency splicing events and novel transcript detection, as the read length often does not cover multiple spliced exon junctions. Further, SRS is sensitive to GC‐content or transcript length‐dependent biases from PCR amplification steps during library preparation. We therefore performed Oxford Nanopore Long‐Read RNA‐seq (LRS) to corroborate SRS analyses, which is less sensitive to false‐positives for specific isoforms. Reads were aligned to GRCm39 and quantified using IsoQuant v3.11.1, identifying 25,093 novel transcripts alongside the reference annotation. We first performed differential isoform expression (DIE) analysis with LRS using edgeR v4.5.0. With aging (aged sham vs adult sham), no isoforms were differentially expressed at *p*‐adj < 0.05 (262 at nominal *p* < 0.05). In adult MOV relative to adult sham, there were 992 and 1029 up‐ and downregulated transcripts, respectively, corresponding to 1820 unique genes. Among the most upregulated transcripts were isoforms of *Mybph* and *Ankrd1* along with downregulation of isoforms of genes in the oxidative phosphorylation pathway (NES = −3.54, *p*‐adj *=* 6.87 × 10^−9^), in line with our previous observations using SRS (Figure 2A,A') (Koopmans et al. 2026). In aged MOV compared to aged sham, only 2 transcripts were significant at *p*‐adj < 0.05 (isoforms of *Mustn1* and *Trem2*), but 638 transcripts were up‐ or downregulated at *p*‐adj < 0.10 (615 unique genes). Cytokine‐cytokine receptor interaction was the most enriched gene set NES = 2.42, *p*‐adj *=* 1.47 × 10^−7^ (Figure 2B,B').

**FIGURE 2:**
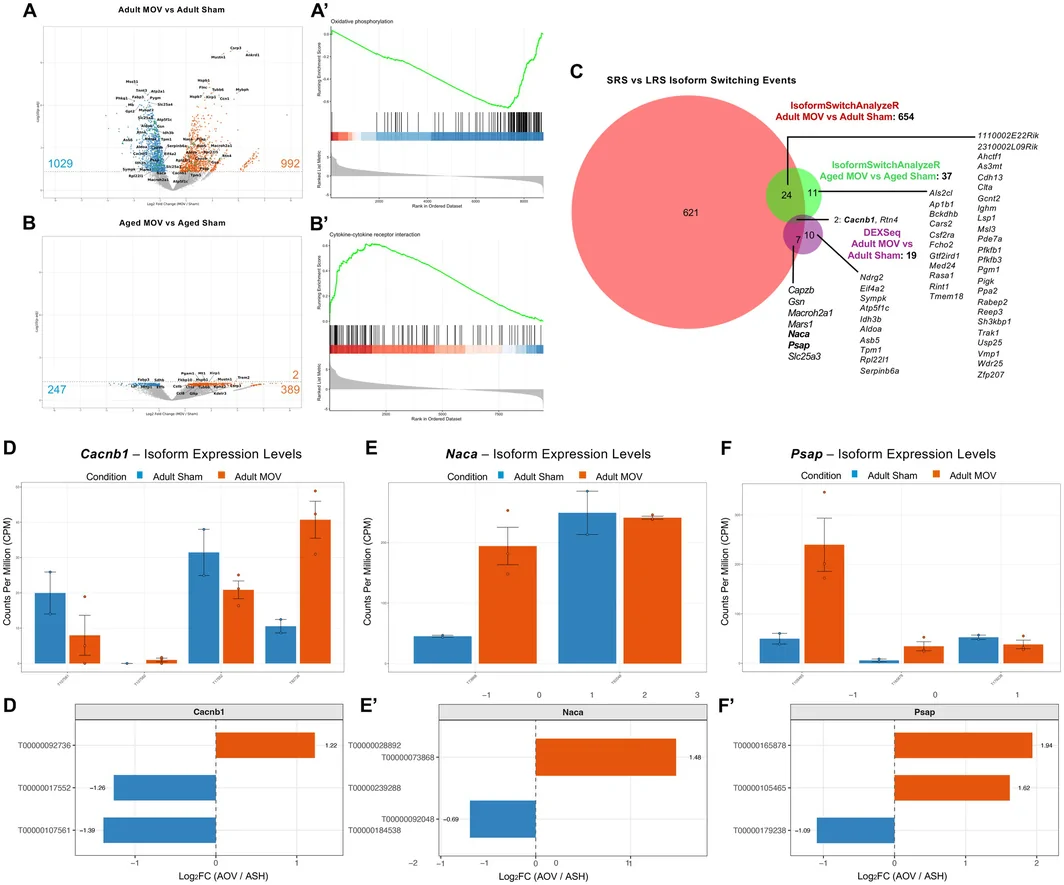
Differential Isoform Expression and Isoform Switching Events in Oxford Nanopore Long‐Read RNA‐Seq. (A) Volcano plot of differentially expressed isoforms with adult MOV, (A') GSEA plot of oxidative phosphorylation pathway. (B) Volcano plot of differentially expressed isoforms with aged MOV, (B′) GSEA plot of cytokine‐cytokine receptor interaction pathway. (C) Venn diagram comparing isoform switching events between SRS and LRS datasets. (D–F) Examples of genes with isoform switching events detected by both SRS and LRS: *Psap* (panel D,D′), *Naca* (Panel E,E'), *Cacnb1* (Panel F,F′). Error bars are mean ± SEM. LRS, *n* = 2–3 replicates (pooled RNA from 2 mice) per condition.

Isoform switching analysis was performed using DEXSeq v1.52.0 (Anders et al. 2012) to distinguish between true isoform switching and coordinated regulation (all isoforms changed in the same direction). *Ndufb8* and *Xirp2* showed highly coordinated responses with upregulation of 6 gene isoforms in adult MOV (Data S1). LRS revealed 19 genes with true isoform switching in adult MOV and none in aged MOV compared to sham conditions (Figure 2C, Figure S2) (Tang et al. 2020). The vastly lower number of genes demonstrating isoform switching with LRS compared to SRS could be due to fewer replicates (*n* = 2–3/group vs *n* = 6‐7/group), potentially limiting statistical power or reduced false discovery rate with greater mapping confidence when assigning reads to specific isoforms. Of these, nine were common isoform switching genes identified by SRS: *Cacnb1, Rtn4, Capzb, Gsn, Macroh2a1, Mars1, Naca, Psap, Slc25a3* (Figure 2C). Although we did not validate via qPCR or at the protein level, concordance between SRS and LRS makes us confident these genes undergo some level of isoform switching. *Cacnb1* (or CaVβ1, Figure 2D,D') has been studied in the context of isoform switching during muscle denervation, regeneration and aging, where embryonic/neonatal isoforms were preferentially expressed over the mature adult isoform as a compensatory mechanism during muscle atrophy (Taylor et al. 2014; Traoré et al. 2019; Vergnol et al. 2025). LRS confirmed a shift in isoform abundance towards the skNAC variant (ENSMUST00000073868) in adult but not aged MOV (Figure 2E,E'). LRS also confirmed greater abundance of the isoform switching of the lysosomal activator protein *Psap* (Prosaposin, Figure 2F,F'), encoding four unique saposin domains (A‐D). PSAP exerts neuroprotective effects during models of oxidative stress and several other diseases (Alzheimer's, trauma, ischemia, diabetes) (Taha et al. 2026). Mammals express two isoforms of *Slc25a3* from alternative usage of exon 3A or 3B. SLC25A3‐A (ENSMUST00000170810) is specific to heart and skeletal muscle, and all other cells express SLC25A3‐B (ENSMUST00000076694) (Fiermonte et al. 1998; Boulet et al. 2018), with the B‐isoform having a 3‐fold higher rate of phosphate transport than the A‐isoform in liposomes from *Escherichia coli* (Taha et al. 2026; Fiermonte et al. 1998). The role of these isoforms in hypertrophic exercise adaptation is not known.

Collectively, our results show aged muscle has a reduced capacity or potentially delayed timing of isoform switching, given the 72 h timepoint of MOV represents the early stage of muscle growth prior to detectable changes in fiber size. A later time point during MOV may provide greater resolution. Additionally, genes with isoform switching were only predicted *in silico*, without further validation at the protein or functional level. Nevertheless, these analyses provide numerous targets which may be explored in future mechanistic studies.

## 5 Methods

### 5.1 Animals

All animal procedures were approved by the University of Arkansas IACUC. Mice were housed in a temperature and humidity‐controlled room, maintained on a 12:12‐h light–dark cycle, and food and water were provided *ad libitum* throughout experimentation. Details on mice and experiments are found in Koopmans et al. (2026). Briefly, at euthanasia (morning, ZT 1–5), animals were first deeply anesthetized with isoflurane and sacrificed via cervical dislocation. Per Koopmans et al. 2026, adult (6–8 months of age) and aged (24 months of age) male human skeletal actin reverse tetracycline transactivator—tetracycline response element histone 2B green fluorescent protein (HSA‐rtTA^+/−^; TRE‐H2B‐GFP^+/−^, or HSA‐GFP) mice were generated and genotyped as previously described by us. HSA‐GFP mice were treated with low‐dose doxycycline (0.5 mg/mL doxycycline in drinking water with 2% sucrose) for 7 days to induce GFP labeling of myonuclei, followed by a washout period (normal drinking water) of at least 7 days.

### 5.2 Synergist Ablation Mechanical Overload Experiment

Synergist ablation mechanical overload (MOV) of the plantaris was performed as previously described by our lab group at a consistent daily interval (ZT 1–5). Briefly, synergist ablation is a surgical procedure that occurs while mice are under anesthesia. The surgery involves making an incision on the posterior aspect of the lower hindlimb, cutting of the Achilles tendon followed by careful removal of 1/3rd of the gastrocnemius–soleus complex while leaving the plantaris muscle and tendon intact. Sham surgery (control) involved all the steps of synergist ablation but no tendon is cut or muscle is removed. Following surgery, mice resumed ambulatory cage activity. Euthanasia was performed 72 h after MOV and plantaris muscles from both limbs were dissected and immediately flash frozen in liquid nitrogen. All muscle was used for downstream molecular analyses across four experimental conditions: adult sham (*n =* 6), adult MOV (*n =* 6), aged sham (*n =* 6), aged MOV (*n =* 7).

### 5.3 RNA Isolation

RNA was isolated from approximately one‐third of each plantaris muscle (~10 mg) using TRIzol Reagent (Sigma‐Aldrich, St. Louis, MO, USA). Tissue was homogenized using zirconia beads and the Fisher Bead Mill (Fisher, Hampton, NH, USA). Following homogenization, RNA was isolated via phase separation by addition of chloroform and then centrifugation. The aqueous phase was transferred to a new sterile tube and further processed on spin columns according to manufacturer instructions using the Direct‐zol Kit (Zymo Research, Irvine, CA, USA). RNA quality was checked on Agilent TapeStation with RNA screentape to confirm quality and purity. RNA integrity number (RIN) was > 7 for all samples (8.3 ± 0.7).

### 5.4 Oxford Nanopore Library Preparation and Sequencing

A minimum of 600 ng of total RNA (pooled from *n* = 2 animals, equal RNA input mass) was used to synthesize PCR‐cDNA libraries using the Direct RNA‐Sequencing Kit (Oxford Nanopore Technologies, ONT; Cat# SQK‐RNA004) according to the manufacturer's instructions. Subsequently, the prepared PCR‐cDNA libraries were quantified and loaded onto a MinION Flow Cell (ONT, Cat# FLO‐MIN004RA) and sequenced (long‐read RNA sequencing, LRS) for 72 h using the MinION Mk1B sequencer (ONT, Cat# MIN‐101B). Base‐calling and FASTQ conversion were performed in super accurate basecalling mode (SUP), set to detect pseudo‐uridine, M6A, M5C, and inosine in all contexts, with MinKNOW version 25.09.16.

### 5.5 Short‐Read Isoform Switching Analyses (IsoformSwitchAnalyzeR)

Previously published high‐depth short‐read RNA‐sequencing (60 million reads/sample) data was used for differential isoform switching analyses (Koopmans et al. 2026). Differentially expressed gene were determined using DESeq2 and defined as *p*‐adj < 0.05 and |Log_2_FC| ≥ 1. Genes from the KEGG spliceosome mmu03040 were log2‐transformed, z‐scored per gene, then scored as mean z of the splicing set minus mean z of expression‐decile‐matched control genes for background correction. Figures were generated in GraphPad Prism version 10.6 for Mac OS X (GraphPad Software, La Jolla, CA) and RStudio.

### 5.6 Long‐Read Isoform Switching Analysis

Sequencing reads were quality controlled using NanoPlot v1.42.0. Reads were aligned to the mouse reference genome (GRCm39 primary assembly) using minimap2 v2.28 with splice‐aware settings (‐ax splice ‐uf ‐k14). The GENCODE vM35 comprehensive gene annotation was used as the reference transcriptome. Transcript‐level quantification was performed using IsoQuant v3.11.1 in complete gene database mode (‐complete_genedb), which quantifies both annotated transcripts and discovers novel isoforms. IsoQuant generated per‐sample transcript count matrices and an extended GTF annotation containing 25,093 novel transcripts alongside the reference annotation. Differential isoform expression analysis was conducted using the edgeR v4.5.0 quasi‐likelihood pipeline in R v4.5.3. Transcripts were filtered to retain those with > 1 count per million (CPM) in at least 2 samples. Library sizes were normalized using the trimmed mean of M‐values (TMM) method. Dispersions were estimated using estimateDisp(), followed by quasi‐likelihood *F*‐tests (glmQLFit and glmQLFTest). Multiple testing correction was performed using the Benjamini‐Hochberg false discovery rate (FDR) method. Transcripts with FDR (*p*‐adj) < 0.05 were considered significantly differentially expressed. Isoform switching analysis was performed using DEXSeq v1.52.0, treating individual transcripts as counting bins within genes. Only genes with two or more detected transcripts were analyzed. The DEXSeq design formula was: ~ sample + exon + condition: exon, with the reduced model ~ sample + exon used for likelihood ratio testing. Transcripts with *p*‐adj  < 0.05 were considered to have significant differential usage. To distinguish between true isoform switching and coordinated regulation, genes with significant isoform switching were classified based on their DIE results. Genes where ≥ 2 isoforms showed significant differential expression (*p*‐adj < 0.05) with opposite‐direction fold changes (one positive logFC, one negative logFC) were classified as exhibiting “true isoform switching.” Genes where all significant isoforms changed in the same direction were classified as showing “coordinated regulation.”

## Author Contributions

P.J.K. and K.A.M. conceived the study. P.J.K., A.I., and A.L.S., performed experiments and/or analysis. C.E.N., A.I., and K.A.M. provided resources. P.J.K. wrote the manuscript draft with input from K.A.M, A.I., and Y.W. All authors reviewed and approved the manuscript.

## Funding

This study was supported by NIH grants AG063944 and AG088465 to K.A.M. This work was performed while K.A.M. was a Glenn Foundation for Medical Research and AFAR Grant for Junior Faculty awardee. This study was also supported by AG093185 to A.I., 1R35GM155433‐01 to C.E.N., and AR081367 to Y.W. Y.W. was also supported by P20GM156679.

## Conflicts of Interest

Y.W. is the founder of MyoAnalytics LLC. The remaining authors have no other competing interests to declare.

## Supporting information


**Figure S1:** (A) Venn diagram comparing number of isoform switching genes with aging vs. differentially expressed genes (DEG, Koopmans et al. 2026; *Advanced Science*), (B–E) Examples of genes with significant isoform switching events, despite showing not significant changes in total gene expression: *Tsc1* (B), *Champ1* (C), *Map4k5* (D), *Hk2* (E). Error bars are mean ± SEM. *n* = 6–7 mice per condition.

**Figure S2:** Plots of all genes identified to have significant isoform switching by LRS. AOV: Adult MOV. ASH: Adult Sham. *n* = 2–3 replicates (pooled RNA from 2 mice) per condition. LRS: Oxford Nanopore Long‐Read RNA‐Sequencing.

**Data S1:** acel70761‐sup‐0003‐DataS1.xlsx.

## Data Availability

Short‐read RNA‐seq data is available at GSE175410. Long‐read RNA‐seq data are available at GSE345121.
