# Supplementary material for: A Hypertrophic Stimulus Drives Age‐Dependent Transcript Isoform Switching With and Without Differential Gene Expression in Skeletal Muscle
Source: Aging Cell. 2026 Oct 14;25(10):e70761. doi: 10.1111/acel.70761 (PMC13669657; doi:10.1111/acel.70761)
Supplement: Supplementary file 1 [file ACEL-25-e70761-s001.pdf]

Figure S1

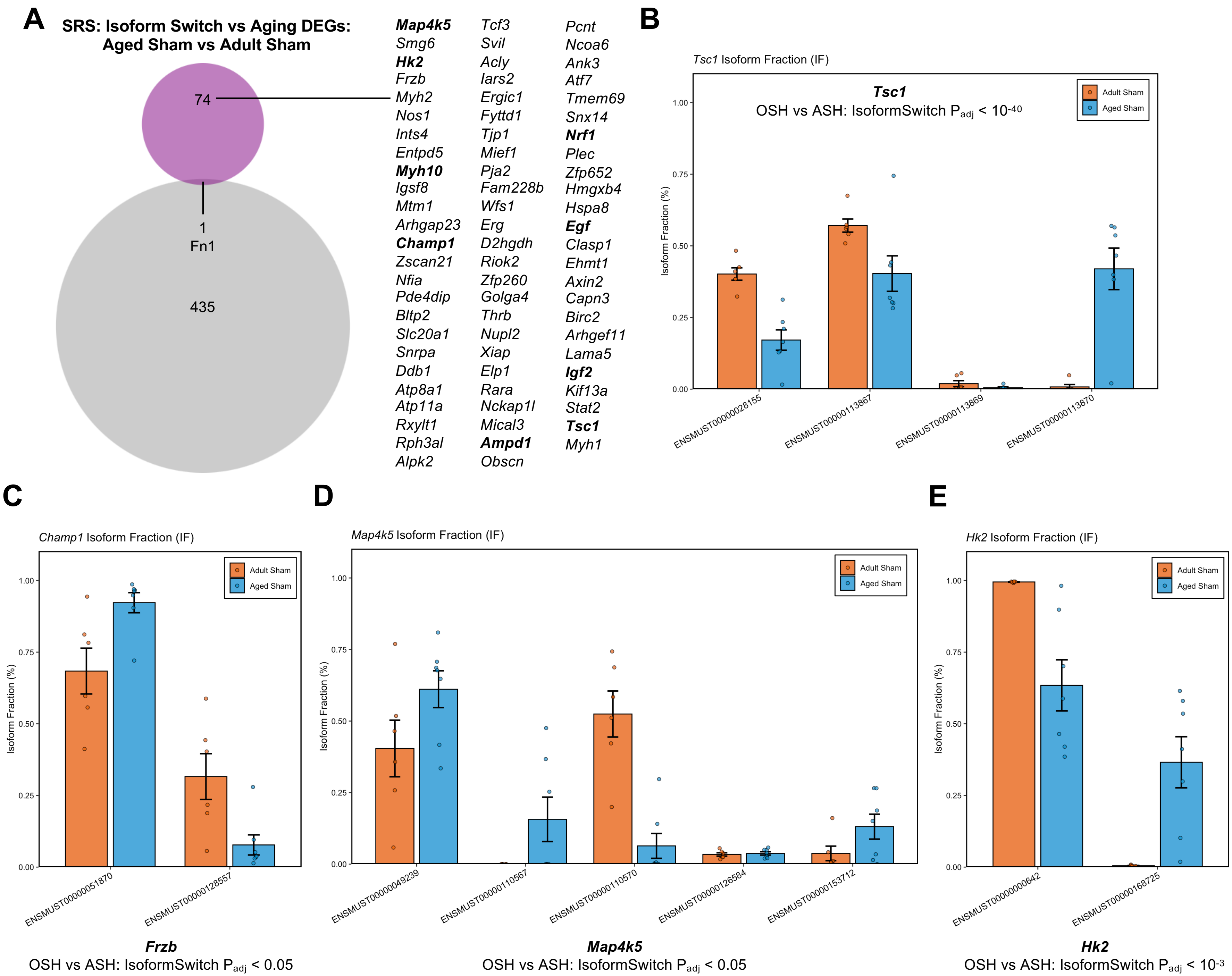
A
SRS: Isoform Switch vs Aging DEGs:
Aged Sham vs Adult Sham
74
1
Fn1
435
Map4k5
Smg6
Hk2
Frzb
Myh2
Nos1
Ints4
Entpd5
Myh10
Igsf8
Mtm1
Arhgap23
Champ1
Zscan21
Nfia
Pde4dip
Bltp2
Slc20a1
Snrpa
Ddb1
Atp8a1
Atp11a
Rxylt1
Rph3al
Alpk2
Tcf3
Svil
Acly
Iars2
Ergic1
Fyttd1
Tjp1
Mief1
Pja2
Fam228b
Wfs1
Erg
D2hgdh
Riok2
Zfp260
Golga4
Thrb
Nupl2
Xiap
Elp1
Rara
Nckap1l
Mical3
Ampd1
Obscn
Pcnt
Ncoa6
Ank3
Atf7
Tmem69
Snx14
Nrf1
Plec
Zfp652
Hmgxb4
Hspa8
Egf
Clasp1
Ehmt1
Axin2
Capn3
Birc2
Arhgef11
Lama5
Igf2
Kif13a
Stat2
Tsc1
Myh1
B
Tsc1 Isoform Fraction (IF)
Tsc1
OSH vs ASH: IsoformSwitch P_adj < 10^-40
Adult Sham
Aged Sham
Isoform Fraction (%)
1.00
0.75
0.50
0.25
0.00
ENSMUST00000028155
ENSMUST00000113867
ENSMUST00000113869
ENSMUST00000113870
C
Champ1 Isoform Fraction (IF)
Adult Sham
Aged Sham
Isoform Fraction (%)
1.00
0.75
0.50
0.25
0.00
ENSMUST00000051870
ENSMUST00000128557
Frzb
OSH vs ASH: IsoformSwitch P_adj < 0.05
D
Map4k5 Isoform Fraction (IF)
Adult Sham
Aged Sham
Isoform Fraction (%)
1.00
0.75
0.50
0.25
0.00
ENSMUST00000049239
ENSMUST00000110567
ENSMUST00000110570
ENSMUST00000126584
ENSMUST00000153712
Map4k5
OSH vs ASH: IsoformSwitch P_adj < 0.05
E
Hk2 Isoform Fraction (IF)
Adult Sham
Aged Sham
Isoform Fraction (%)
1.00
0.75
0.50
0.25
0.00
ENSMUST00000000642
ENSMUST00000168725
Hk2
OSH vs ASH: IsoformSwitch P_adj < 10^-3
