# Supplementary material for: A Hypertrophic Stimulus Drives Age‐Dependent Transcript Isoform Switching With and Without Differential Gene Expression in Skeletal Muscle
Source: Aging Cell. 2026 Oct 14;25(10):e70761. doi: 10.1111/acel.70761 (PMC13669657; doi:10.1111/acel.70761)
Supplement: Supplementary file 2 [file ACEL-25-e70761-s002.pdf]

Figure S2

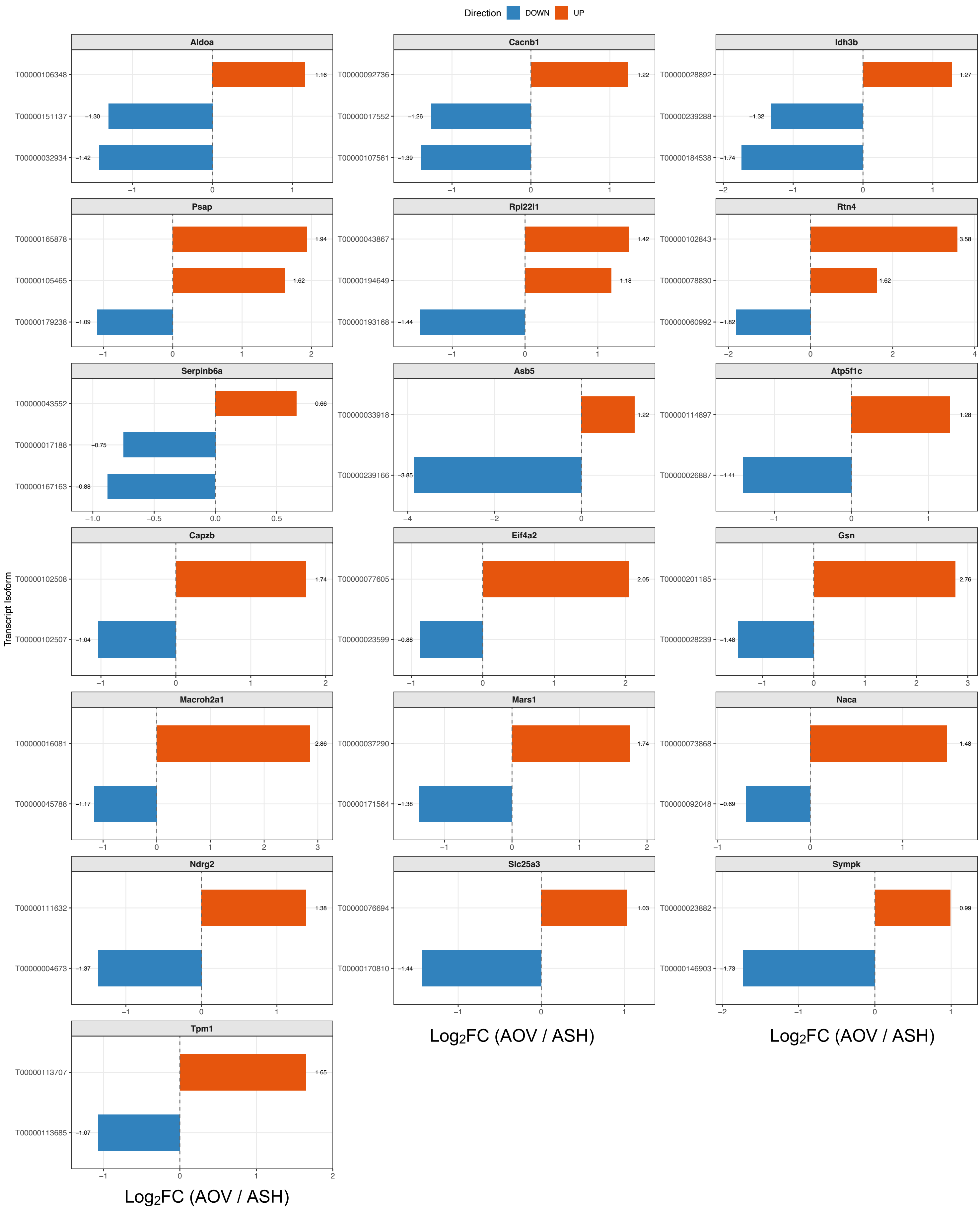

Direction
DOWN
UP
Transcript Isoform
Aldoa
T00000106348
T00000151137
T00000032934
1.16
−1.30
−1.42
Cacnb1
T00000092736
T00000017552
T00000107561
1.22
−1.26
−1.39
Idh3b
T00000028892
T00000239288
T00000184538
1.27
−1.32
−1.74
Psap
T00000165878
T00000105465
T00000179238
1.94
1.62
−1.09
Rpl22l1
T00000043867
T00000194649
T00000193168
1.42
1.18
−1.44
Rtn4
T00000102843
T00000078830
T00000060992
3.58
1.62
−1.82
Serpinb6a
T00000043552
T00000017188
T00000167163
0.66
−0.75
−0.88
Asb5
T00000033918
T00000239166
1.22
−3.85
Atp5f1c
T00000114897
T00000026887
1.28
−1.41
Capzb
T00000102508
T00000102507
1.74
−1.04
Eif4a2
T00000077605
T00000023599
2.05
−0.88
Gsn
T00000201185
T00000028239
2.76
−1.48
Macroh2a1
T00000016081
T00000045788
2.86
−1.17
Mars1
T00000037290
T00000171564
1.74
−1.38
Naca
T00000073868
T00000092048
1.48
−0.69
Ndrg2
T00000111632
T00000004673
1.38
−1.37
Slc25a3
T00000076694
T00000170810
1.03
−1.44
Sympk
T00000023882
T00000146903
0.99
−1.73
Tpm1
T00000113707
T00000113685
1.65
−1.07
Log2FC (AOV / ASH)
Log2FC (AOV / ASH)
Log2FC (AOV / ASH)
